# Epidemiology and critical care management of patients admitted after intentional self-poisoning

**DOI:** 10.1186/cc13262

**Published:** 2014-03-17

**Authors:** GP Misselbrook, N Sudhan

**Affiliations:** 1 Norfolk and Norwich University Hospital NHS Foundation Trust, Norwich, UK

## Introduction

Intentional self-poisoning is one of the most common presentations to acute medical units across the UK [1]. To our knowledge no studies have been published on incidence of admissions to critical care in England after overdose. Our aim was to investigate the epidemiology, clinical features and outcomes of patients admitted to critical care after intentional self-poisoning to establish patterns in our community.

## Methods

We performed a retrospective data collection using our critical care database 'Metavision' to select all patients admitted with a diagnosis of 'overdose'. Records were scrutinised to collect information on patient demographics, clinical features and medical management.

## Results

Thirty-eight patients (male:female ratio 1:1.53) were admitted to critical care over a 1-year period (September 2011 to 2012). This represented 2.45% of the total admissions to critical care during the same period. The sample had a significantly younger median age (45 years) than the standard patient population in critical care (68 years) during the same period (*P *< 0.0001). Despite the young age and paucity of comorbidities, there was no difference in length of stay between overdose patients (2.0 days) and all other patients on critical care (1.58 days, *P = *0.3). The median number of agents ingested was three (1 to 7) with 84.2% ingesting ≥2 agents. Hypnotics and antidepressants made up 45% of the agents ingested. A total 92.1% of the sample was admitted out-of-hours or at weekends. Fifty per cent had a past history of overdose, 25% had a history of alcohol misuse. A total of 79% of patients were referred to critical care due to a low conscious level but only 50% required IPPV and 20% received vasopressors/inotropes. The mortality rate was 2.6%, with one further death 6 months after discharge due to alcoholic liver disease. Estimated financial cost was £80,555 or £2,119 per patient (57 level 3 bed-days, 35 level 2 bed-days).

## Conclusion

Intentional self-poisoning mortality rates are low in spite of the number of patients admitted. Despite the young age of patients and lack of comorbidities, their length of stay is similar to the average length of stay for all patients admitted to the unit, representing a significant financial cost. Self-poisoning requiring critical care support is more common out-of-hours when less senior expertise is available. Education of junior doctors into management of overdose is therefore vital to ensure the early identification and appropriate treatment of these patients.
